# Sickle Cell Nephropathy and Associated Factors among Asymptomatic Children with Sickle Cell Anaemia

**DOI:** 10.1155/2020/1286432

**Published:** 2020-08-14

**Authors:** Uchenna Modestus Nnaji, Christian Chukwukere Ogoke, Henrietta Uche Okafor, Kingsley I. Achigbu

**Affiliations:** ^1^Department of Paediatrics, Federal Medical Centre, Owerri, Nigeria; ^2^Department of Clinical Neurophysiology & Child Neurology, Mother Healthcare Diagnostics & Hospital, Owerri, Nigeria; ^3^Department of Paediatrics, University of Nigeria, Enugu Campus & University of Nigeria Teaching Hospital, Ituku-Ozalla, Enugu, Nigeria

## Abstract

**Background:**

Sickle cell nephropathy (SCN) is a serious complication of sickle cell anaemia (SCA) with asymptomatic onset in childhood and possible progression to chronic kidney disease (CKD). In Southeast Nigeria, few studies have evaluated renal function in paediatric SCA patients for early detection of renal impairment and early intervention to reduce morbidity and mortality. Therefore, this study evaluated the renal function of paediatric SCA patients in a steady state based on glomerular filtration rate and urinalysis findings (proteinuria and haematuria).

**Methods:**

A cross-sectional study of consecutively recruited sixty haemoglobin SS (HbSS) children in a steady state and sixty age- and sex-matched haemoglobin AA (HbAA) controls aged 2–18 years was done. Renal function of HbSS subjects was evaluated using estimated glomerular filtration rate (eGFR) which was compared with healthy HbAA subjects. The prevalence of significant proteinuria and haematuria, its association with eGFR, and the effect of past sickle cell crisis (in the preceding 24 months) on renal function were also evaluated.

**Results:**

Mean eGFR was significantly higher in HbSS subjects than in the HbAA subjects (*p* = 0.001) and decreased with age. Significant proteinuria and haematuria were more prevalent in the HbSS group (3.4% and 6.7%, respectively) compared to the HbAA subjects (0% and 0%, respectively) (*p* = 0.496 and 0.119, respectively). No significant association was observed between eGFR and proteinuria (*p* = 1.000) or haematuria (*p* = 1.000). There was a positive correlation between eGFR and frequency of past painful crisis that required hospitalization (*r* = 0.138, *p* = 0.295) and between eGFR and frequency of blood transfusion (*r* = 0.679, *p* ≤ 0.001).

**Conclusions:**

Asymptomatic paediatric HbSS (SCA) patients had higher mean eGFR indicating an increased risk of nephropathy. There was no association between eGFR and proteinuria or haematuria. Frequent sickle cell crises especially one requiring transfusion were positively correlated with hyperfiltration.

## 1. Introduction

Sickle cell anaemia (SCA) is a monogenetic chronic anaemia and the most prevalent genetic disease in Africa. Worldwide, about 300,000 babies are born with sickle cell disease (SCD) each year with 75% of them in sub-Sahara Africa [1]. Nigeria alone accounts for more than 150,000 new births with SCD each year and has a prevalence of homozygous sickle cell disease (HbSS) or SCA of 2-3% [1].

SCN constitutes 0.11% of end-stage renal disease (ESRD) reported in the United States renal data survey (USRDS), 93% of which were in African Americans, and SCA was documented as a risk factor for ESRD [2]. A prospective study by Powars et al. [3] showed that severe anaemia, hypertension, proteinuria, and microscopic haematuria were significant predictors of chronic renal failure in SCN. With improvement in the medical management of SCA, the survival rates of the patients have steadily improved and CKD in SCA will likely play a greater role in morbidity and mortality in the future [4].

SCN begins in childhood and is now a well-characterised entity with specific manifestations, risk factors, and prognosis [5]. SCN encompasses all the structural, clinical, and laboratory or functional changes that occur in patients with SCA due to renal involvement resulting from chronic sickling of the abnormal red blood cells [5]. It involves damage to multiple structures within the kidney including the glomeruli within the renal cortex, the renal tubules, and vasa recta within the hypoxic, hyperosmolar renal medulla. Therefore, SCN includes glomerular abnormalities (sickle cell glomerulopathy) and renal tubular abnormalities (sickle cell tubulopathy) with variable clinicopathologic presentation such as renal ischaemia, infarction, papillary necrosis, haematuria, hyposthenuria, proteinuria, impaired urinary acidification, nephrotic syndrome, and renal failure [5, 6].

Glomerular hyperfiltration is common in children with SCA, and the glomerular hypertrophy that develops leads to glomerulosclerosis and chronic kidney disease (CKD) [6]. Hyperfiltration assessed by estimated GFR is found in 51%, proteinuria (albuminuria) in 59%, and chronic renal failure in 7% of SCA patients compared with only 5%, 7%, and 2%, respectively, in young HbSC patients [2]. Proteinuria is more commonly encountered in homozygous (HbSS) SCD than in other haemoglobinopathies and is both an early sign and a predictor of progression of SCN [7].

There is dearth of studies carried out in South-Eastern Nigeria on renal function in paediatric SCA patients using glomerular filtration rate (GFR) and urinalysis [8–10]. This study evaluated the renal function based on glomerular filtration rate and urinalysis findings (proteinuria and haematuria) for the early detection of renal impairment in SCA. Such early detection and prompt institution of affordable treatment/interventions will contribute to the prevention of CKD and reduce the morbidity and mortality from SCN.

## 2. Materials and Methods

This study was carried out at the Federal Medical Centre (FMC) Owerri, a tertiary health care facility for Imo state and its environs in Southeast Nigeria. The paediatric sickle cell clinic (PSCC) is one of its eight subspecialty clinics and runs once a week (every Monday). On the average, 6-8 patients with SCA are seen on each clinic day. The children outpatient clinics (CHOP) run five times a week (Mondays to Fridays) and attends to an average of 75 patients per day. The PSCC and CHOP are both run by a clinical team of consultants, residents, and house officers. The FMC Owerri has modern laboratory facilities with qualified laboratory personnel and is open for services all day.

This was a descriptive cross-sectional hospital-based study. The study populations were HbSS patients in a steady state aged 2-18 years who attended PSCC of FMC Owerri between June 2016 and September 2016 and their age-/sex-matched HbAA subjects that attended CHOP during the same period. SCA was confirmed with haemoglobin electrophoresis, and all had HbSS genotype. Haemoglobin electrophoresis done for controls also confirmed their HbAA genotype before they were included in the study. The HbAA group was mainly children on follow-up at CHOP after recovery from common childhood illnesses like respiratory tract infection, uncomplicated malaria, and cutaneous fungal infections and those presenting with surgical cases like hernia. HbSS and HbAA subjects who met the inclusion criteria were consecutively recruited from PSCC and CHOP, respectively. The researcher attended the clinics until required sample size was reached.

### 2.1. Inclusion Criteria

#### 2.1.1. HbSS Subjects

(1) All children with SCA in a steady state aged 2-18 years who gave their assent (where necessary) and also whose parents/guardian consented to the study. Subjects in a steady state were those that met the following criteria [11, 12]:
  - Absence of fever at presentation and in the preceding four weeks
  - Absence of skeletal and/or abdominal pain at presentation and in the preceding four weeks
(2) Those receiving no other medication apart from routine folic acid, vitamin B complex, and proguanil

#### 2.1.2. HbAA Subjects

1. Children with haemoglobin AA matched for sex and age with the HbSS subject whose parents/guardians gave informed consent as well as assent from subjects

### 2.2. Exclusion Criteria

#### 2.2.1. HbSS Subjects

1. Subjects with preexisting renal disease
2. Subjects with overt clinical features of urinary tract infection (UTI)
3. Adolescent female subjects menstruating at the time of the study
4. Subjects with gross haematuria
5. Subjects not in a steady state

#### 2.2.2. HbAA Subjects

1. Patients with known renal disease or clinical features of UTI
2. Female subjects menstruating at the time of study

### 2.3. Study Procedure/Data Collection

Relevant history was obtained including number of hospital admissions following painful crisis, number of blood transfusion in the past 2 years, and urogenital system review to rule out UTI/preexisting renal disease. General physical examination and examination of systems with particular attention to urogenital and cardiovascular systems were done, and findings suggestive of SCA and renal disease were sought for such as asthenia/sickle cell habitus, pallor, jaundice, and facial swelling/puffiness.

The following anthropometric indices were assessed: weight (kg), height (cm), body mass index (BMI) (kg/m^2^), and body surface area (BSA) (m^2^). The weight and height were measured using a combined measuring instrument health weighing scale and stadiometer (RGZ 160 model manufactured by Globe Medical England) with respective sensitivities of 0.1 kg and 0.1 cm. The scale was set at zero before each measurement on each day. Blood pressure (BP) was measured using mercury sphygmomanometer (Accoson brand) in combination with open bell of a Littman classic paediatric and adult stethoscope. Appropriate-sized BP cuffs were used (cuff bladder width to length ratio of at least 1 : 2 covering at least 40% of the right arm and bladder length covering 80-100% of the right arm).

Four millilitres (4 ml) of venous blood was collected by venepuncture, 2 ml placed in a plain bottle (free of anticoagulant) for serum creatinine measurement, and the remaining 2 ml placed in an ethylene diamine tetra-acetic acid (EDTA) tube for haemoglobin electrophoresis and haemoglobin concentration estimation. About 15 ml of first morning 23232midstream urine sample was collected by the caregiver (or the subject for an older child) in a labelled sterile universal container. Caregiver/subject was instructed on how to appropriately collect urine and to bring the sample to the researcher within 2 hours of collection (as recommended by manufacturers of the urinalysis dipstick). Urinalysis was done by the researcher with Multistix 10SG dipstick (Made in Germany) for testing ten different parameters: protein, blood, specific gravity, pH, nitrite, leucocyte esterase, glucose, urobilinogen, bilirubin, and ketone. Significant proteinuria or haematuria was taken as proteinuria or haematuria of ≥2+ on dipstick, respectively [13].

The blood sample for serum creatinine analysis was allowed to clot and centrifuged at 3000 revolutions per minute for five minutes the same day the sample was collected. The serum obtained was then dispensed into another plain tube and stored in the freezer at -20°C until serum creatinine was assayed with a Baur UV-1200 autoanalyser using the alkaline picrate (Jaffe) method which is based on a complex formation between alkaline picrate and creatinine. Haemoglobin electrophoresis using cellulose acetate paper at an alkaline pH of 8.6 was done for each subject to confirm the haemoglobin genotype. Also, the haemoglobin concentration was estimated.

Estimated glomerular filtration rate (eGFR) was calculated for each subject using the original Schwartz method with the result of serum creatinine (mg/dl) and the measured height (cm) taking into consideration the different values for the constant “*K*” for each age group and gender: 0.55 for children aged 1-13 years and adolescent females above 13 yrs. “*K*” is 0.70 for adolescent boys above 13 yrs [14].

The renal function of each participant was determined using the estimated GFR and classified according to estimated GFR categories of KDIGO guidelines [15]. Estimated GFR was categorized into high (hyperfiltration), normal, and mildly decreased groups. The high and normal eGFR belong to GFR category G1: eGFR ≥ 90 ml/min/1.73 m^2^ [15]. Hyperfiltration is defined as an eGFR value greater than two standard deviations from the mean eGFR obtained from the control subjects (HbAA subjects) while normal eGFR is an eGFR value not more than two standard deviations from the mean eGFR of the HbAA subjects [16]. Mildly decreased eGFR belong to GFR category G2, eGFR 60-89 ml/min/1.73 m^2^ [15].

## 3. Data Analysis

The data was collated, sorted, and entered into the computer. Analysis of sociodemographic, anthropometric, and laboratory data was done using the Statistical Package for the Social Sciences (SPSS) version 20.0. Continuous variables such as age, height, weight, BMI, BSA, and blood pressure were analysed and expressed as mean and standard deviation. Frequency table was used to show the frequency of past painful crisis and blood transfusion. Comparison of means such as height, weight, BMI, blood pressure, and eGFR of HbSS and HbAA subjects was done using Student's *t*-test while analysis of variance (ANOVA) was used to compare eGFR across age groups. Categorical variables were analysed using chi-squared (*χ*^2^) test and Fisher's exact test. Pearson correlation coefficient was used to ascertain any relationship between eGFR and frequency of past crises. Significant level was taken at *p* value less than 0.05 (at 95% confidence interval).

## 4. Results

### 4.1. General Characteristics of the Study Population

One hundred and twenty subjects aged 2–8 years participated in this study comprising 60 patients with sickle cell anaemia (SCA) (HbSS genotype) and 60 age- and sex-matched subjects with HbAA genotype. The SCA patients had a mean age of 9.0 ± 4.2 years while the mean age of the HbAA subjects was 9.1 ± 4.2 years. There was no significant difference in the mean age between the HbSS and HbAA subjects. There were 31 (51.7%) males and 29 (48.3%) females in each group, giving a M : F ratio of 1.07 : 1.

### 4.2. Anthropometric and Blood Pressure Measurements of the Study Population

The mean body weight, height, BMI, and BSA were found to be lower in HbSS subjects compared to the HbAA group though the difference was not statistically significant. Similarly, the mean systolic BP and diastolic BP were lower in HbSS subjects compared to the HbAA group. The difference in mean blood pressure also was not statistically significant as shown in Table 1.

### 4.3. Specific Characteristics of the HbSS Subjects

The median age of diagnosis of SCA was 2 years with a range of 0.5-15 years. Nineteen (31.7%) of the HbSS subjects have never had painful crisis necessitating hospital admission in the past 24 months while 34 (56.7%) did not receive blood transfusion in the past 24 months. The mean frequency of painful crisis and blood transfusion in the past 24 months was 1.22 ± 0.14 and 0.7 ± 0.1, respectively.

## 5. Laboratory Parameters

### 5.1. Haemoglobin Concentration and Creatinine

The haemoglobin concentration (Hb) of the HbSS subjects ranged from 6.3 to 9.3 g/dl with a mean of 7.5 ± 1.2 g/dl. This was significantly lower than that of the HbAA subjects with a mean value 11.3 ± 2.1 g/dl and ranged from 9.3 to 15.0 g/dl, *p* < 0.001. There was a significant difference in the mean creatinine values between the HbSS and HbAA subjects (0.5 ± 0.2 mg/dl vs. 0.6 ± 0.2 mg/dl; *p* = 0.034) as shown in Table 2.

### 5.2. Renal Function of the Study Population

The calculated mean eGFR was higher in HbSS subjects (146.5 ± 31.1 ml/min/1.73 m^2^) than the HbAA group (128.7 ± 18.5 ml/min/1.73 m^2^), and this was statistically significant (*p* ≤ 0.001). None of the study subjects had eGFR < 60 ml/min/1.73 m^2^. The categories of eGFR are shown in Table 3, and there was a significant difference in the categories of eGFR of the study population; hyperfiltration was significantly higher in the HbSS subjects (*p* = 0.001) compared to the HbAA group.

### 5.3. Mean Estimated GRF according to Age Group and Gender

There was no significant difference in these means (*t* − test = −1.404, *p* value = 0.166) as shown in Table 4. But when the mean eGFR was compared across the age groups, the difference was significantly higher in HbSS subjects aged 1-6 years (*p* value = 0.024 using ANOVA) while in the HbAA subjects, there was no significant difference in the mean eGFR across age groups (*p* value = 0.328).

### 5.4. Prevalence of Significant Proteinuria and Haematuria in the Study Population

Among the 60 HbSS subjects studied, 2(3.4%) and 4(6.7%) had significant proteinuria and haematuria, respectively, while none of the HbAA subjects had significant proteinuria or haematuria as shown in Table 5.

### 5.5. Association between Estimated GFR and Proteinuria and Haematuria

Table 6 shows the association between the eGFR and proteinuria as well as haematuria. There was no significant association between estimated GFR and proteinuria (*p* = 1.000) as well as eGFR and haematuria (*p* = 1.000).

### 5.6. Renal Function and Frequency of Past Crisis in HbSS Subjects

Positive correlations were found between estimated GFR and frequency of past painful crisis and blood transfusion. There was also a weak negative correlation between GFR and haemoglobin concentration as shown in Table 7.

## 6. Discussion

The early detection of the asymptomatic phase of sickle cell nephropathy (SCN) is critical to ameliorating its associated morbidity and mortality. This study examined the renal function of HbSS children in a steady state in a tertiary health facility. The mean eGFR of 146.5 ± 31.1 ml/min/1.73 m^2^ in HbSS subjects is comparable to findings by Madu et al. [17] (150 ± 74.8 ml/min/1.73 m^2^) in Enugu South-East Nigeria, Abhulimhen-Iyoha et al. [18] (148.96 ± 23.2 ml/min/1.73 m^2^) in Benin City South-South Nigeria, and Bodas et al. [4] (140 ml/min/1.73 m^2^) in the USA. The mean eGFR in HbSS subjects in this study was significantly higher than that of age- and sex-matched HbAA subjects (128.7 ± 18.5 ml/min/1.73 m^2^). This is similar to findings by Oyinade [19] in Lagos South-West Nigeria and Aloni et al. [20] in the Democratic Republic of Congo (DRC) but differs from other studies [8, 10, 21] in Nigeria that did not find any significant difference between the two groups. The significantly higher eGFR observed in HbSS subjects in this study may be due to the fact that SCA is a risk factor for renal impairment. The ischaemic renal medullary injury following sickling of red blood cells leads to compensatory hypersecretion of prostaglandins and other vasodilators, which leads to glomerular hyperfiltration [5]. Redistribution of renal blood to cortical vessels due to damage to renal medulla vasculature following repeated ischaemia and infarction could also explain the glomerular hyperfiltration [5]. Researchers have attributed the observed varied results in renal function both in local and international studies to differences in methodology, *β*_6_ globin haplotype (genetics), and frequency of clinical events/crisis [3, 4, 22]. Thus, the observed difference noted between this study and the other local studies [8, 19, 21] may be due to the methodology: the index study included more younger age category and used a more inclusive age group (2-18 years) while the other studies used a narrower age category. Again, this study used creatinine-based Schwartz method of estimating GFR unlike the cumbersome and frequently inaccurate 24-hour urine sample used by the other studies to measure creatinine clearance (with exception of Okoro and Onwuamaeze [10] who used creatinine-based Schwartz method).

The various *β*_6_ globin haplotypes have been associated with varying degrees of renal involvement [3, 22]. This present study found significant glomerular hyperfiltration in 16.7% of HbSS patients versus 0% in the HbAA group while Aloni et al. [20] in DRC reported significant glomerular hyperfiltration in 30.8% of HbSS subjects against 6.1% in the HbAA group. Both studies point to a higher prevalence of hyperfiltration in HbSS subjects. However, the lower prevalence in the index study suggests the possibility of mild renal pathology in Nigerian children with SCA known to have the Benin haplotype (associated with mild disease) compared to DRC with Bantu globin haplotype which is associated with severe renal disease. Also, this study used eGFR values > 2 SD above the normal mean for age as cutoff for glomerular hyperfiltration while Aloni et al. [20] used eGFR values > 140 ml/min/1.73 m^2^ which may have increased their observation.

The resultant glomerular hyperfiltration injury (repeated renal medullary ischaemia and infarction) from recurrent VOC/painful crises worsens with increasing age leading to glomerular hypertrophy and glomerulorsclerosis and a progressive decline in renal function/eGFR. More so, the frequent use of analgesics like NSAIDS in SCA patients (to control pain) contributes to renal damage/reduces GFR over time since NSAIDS can independently induce interstitial nephropathy [5]. There was a statistically significant negative correlation between eGFR and age among HbSS subjects in this study. HbSS subjects among age group 1-6 years had the highest eGFR while the lowest eGFR was found among 13-18-year age group. This is similar to findings by other workers [21, 23] and suggests a progressive decline in renal function among SCA patients. None of the studied HbSS subjects (and also the HbAA group) had eGFR < 60 ml/min/1.73 m^2^. This is similar to findings by other workers [4, 10, 20, 21]. SCA subjects have high eGFR early in life, and a longer period is required to get a decline in eGFR. Majority of the study population with mildly decreased eGFR were >10 years old.

Infrequent painful crisis requiring hospitalization, stable haemoglobin level, and young age are documented low-risk factors for proteinuria [5, 24]. The prevalence of significant proteinuria (3.4%) amongst HbSS subjects in this study is similar to report by Osei-Yeboah and Rodrigues [25] in Ghana and Stallworth et al. [26] in the USA with a prevalence of 2.8% and 3.2%, respectively. It is lower than the report by Ugwu and Eke [13] in Port Harcourt, Aloni et al. [20] in DRC, and Falk et al. [7] in the USA with a prevalence of 7%, 6.2%, and 26.5%, respectively. The HbSS subjects in this study were diagnosed early and had fewer painful crises that needed hospitalization and hence reduced risk of renal damage, and this may explain the observed lower prevalence when compared to other workers [7, 13, 20]. Also, other studies [7, 20] included proteinuria of 1+ as significant while this study used proteinuria of ≥2+ as significant and this may have increased the prevalence of significant proteinuria in their report. Again, 81% of the subjects in Falk et al.'s [7] study were older age (≥17 years) and this may explain the high prevalence of 26.5% in their study. The young age group of subjects in the index study could also account for the low prevalence in the study.

It has been proposed that haematuria is common in SCA since the recurrent sickling of the red blood cells leads to capillary congestion/necrosis of the renal papilla and extravasation of blood into urine. The prevalence of significant haematuria (6.7%) in HbSS subjects in this study is similar to findings by Stallworth et al. [26] and Ugwu and Eke [13] with prevalence rates of 6.3% and 11%, respectively, but higher than findings by Osei-Yeboah and Rodrigues [25] and Anigilaje and Adedoyin [27] with a prevalence of 2.6% and 2.6%, respectively. The reason for the observed difference in the prevalence of haematuria between this work and that of other workers [25, 27] is not easily discerned as similar methodology was used. Haematuria occurred more in the older age group (the youngest child with significant haematuria in this study was 6 years). This is similar to a report in other studies [13, 25, 27]. Also, there was no statistically significant difference in the prevalence of haematuria among gender. The pathophysiology of haematuria in SCA is similar in both males and females, and gender disparity is not expected [27].

There was no significant association between eGFR and proteinuria as well as eGFR and haematuria in this study. The mean eGFR of HbSS subjects with significant proteinuria was lower (though still within normal range) than those with insignificant proteinuria but the difference was not statistically significant. This is similar to findings by other workers [18, 24, 28] while some studies [13, 25, 27] did not assess any association between eGFR and haematuria. In this study, HbSS subjects with haematuria had normal eGFR. This may be explained by the fact that the pathology of haematuria in sickle cell disease is generally benign and self-limiting [5].

It is expected that the higher the frequency of painful crisis, the higher the level of compensatory vasodilator prostaglandins produced and increased eGFR until at a point when the continual ischaemic renal injury will lead to loss of medullary mass/function and decline in prostaglandin levels and eGFR. In this study, a weak positive correlation was found between eGFR and frequency of hospitalized past painful crisis. Similar to the index study, Bodas et al. [4] and Madu et al. [17] did not find any statistically significant correlation between eGFR and frequency of past painful crisis in their study. This may be due to few past history of painful crisis obtained from the subjects in this study. Other workers [9, 29] showed a reduction in eGFR during VOC and hyperhemolytic crisis in HbSS subjects with reversal to normal following recovery (steady state). However, they did not correlate eGFR with the frequency of past painful crisis or anaemic crisis. Frequent sickle cell crises especially one requiring transfusion were positively correlated with eGFR in this study. The study also showed that those with lower haemoglobin concentration had a higher GFR and at risk of SCN though the correlation was not strong. Severe anaemia requiring blood transfusion in HbSS subjects is due to haemolysis, and its role in pathophysiology of SCN is well established [3]. It has been shown that prevention of anaemia by blood transfusion can actually reduce glomerular hyperfiltration and progressive renal injury [30].

### 6.1. Limitations

A single-centred hospital-based study would limit the generalization of findings of this study. The preferred method to assess proteinuria is doing spot urine albumin to creatinine ratio. This study used the conventional urine dipstick which has low sensitivity for microalbuminuria and hence may have underestimated the true prevalence of proteinuria and SCN in the study population.

### 6.2. Conclusions

Abnormally high eGFR indicating an increased risk of nephropathy and a low prevalence of significant proteinuria and haematuria were seen in asymptomatic paediatric SCA patients in our locality. There was no association between eGFR and proteinuria or haematuria. Frequent sickle cell crises especially one requiring transfusion were positively correlated with hyperfiltration. Therefore, regular monitoring of renal function in asymptomatic paediatric SCA patients and a comprehensive healthcare management protocol to prevent crises (painful and anaemic) are recommended.

## Figures and Tables

**Table 1:** Anthropometric and Blood Pressure Measurements of the Study Population.

	HbSS mean ± SD	Range	HbAA mean ± SD	Range	*t*-test	*p* value
Weight (kg)	28.6 ± 13.4	11.0–63.0	31.3 ± 12.6	13.00–65.0	-1.172	0.244
Height (cm)	129.5 ± 19.7	86.0–168.0	132.8 ± 19.9	92.0–163.0	-0.899	0.371
BMI (kg/m^2^)	16.1 ± 2.9	12.0–28.4	17.0 ± 2.4	13.0–24.8	-1.910	0.059
BSA (m^2^)	1.0 ± 0.3	0.51-1.68	1.05 ± 0.3	0.6–1.70	-0.865	0.389
Blood pressure						
SBP (mmHg)	96.9 ± 7.5	80.0–120.	99.5 ± 8.2	80.0-120.0	-1.794	0.075
DBP (mmHg)	57.2 ± 8.1	40.0–80.0	59.7 ± 9.3	40.0–85.0	-1.995	0.120

**Table 2:** Mean Hb and creatinine of the study population.

Laboratory parameters	HbSS		HbAA		*t*-test	*p* value
	Mean ± SD	Range	Mean ± SD	Range		
Hb	7.5 ± 1.2	6.3–9.3	11.3 ± 2.1	9.3–15.0	-22.408	0.000
Creatinine	0.5 ± 0.2	0.2–0.9	0.6 ± 0.2	0.4–1.2	-2.147	0.034

**Table 3:** Categories of eGFR.

eGFR category	HbSS 𝑛 =60 (%	HbAA 𝑛 =60 (%)	Total 𝑛 =120	Statistical test	*p* *value*
Hyperfiltration	10 (16.7)	0 (0.0)	10 (8.3)		
Normal	47 (78.3)	58 (96.7)	105 (87.5)	Fishers	0.001
Mildly decreased	3 (5.0)	2 (3.3)	5 (4.2)		

Key: hyperfiltration **(**high) and normal: eGFR category G1 (≥90 ml/min/1.73 m^2^), mildly decreased: eGFR category G2 (60-89 ml/min/1.73 m^2^). None of the study population was in categories G3a–G5 (<60 ml/min/1.73 m^2^).

**Table 4:** Mean eGFR of the study population according to age group and gender.

Age group (years)	eGFR		*t*-test	*p* value
	Mean ± SD (ml/min/1.73 m^2^)			
	HbSS (*n* = 60)	HbAA (*n* = 60)		
1-6	161.3 ± 28.1	128.5 ± 18.6	4.057	≤0.001^∗^
7-12	146.7 ± 32.9	130.3 ± 17.3	2.330	0.023^∗^
13-18	127.9 ± 19.6	126.2 ± 21.3	0.219	0.828
Gender				
Male	147.7 ± 28.5	125.7 ± 19.0	3.650	0.001^∗^
Female	145.3 ± 34.1	132.4 ± 17.4	1.763	0.004^∗^

**Table 5:** Prevalence of significant proteinuria and haematuria in the study population.

Urinalysis findings	HbSS *n* = 60 (%)	HbAA *n* = 60 (%)	Total *n* = 120 (%)	Statistical test	*p* value
Proteinuria					
Insignificant	58 (96.6)	60 (100.0)	118 (98.4)	Fishers'	0.496
Significant	2 (3.4)	0 (0.0)	2 (1.6)		
Haematuria					
Insignificant	56 (93.3)	60 (100.0)	116 (96.7)	Fisher's	0.119
Significant	4 (6.7)	0 (0.0)	4 (3.3)		

**Table 6:** Association between estimated GFR and proteinuria and haematuria among SCA patients.

Variable	Estimated GFR					
	Hyperfiltration	Normal	Mildly decreased	Total	Statistical test	*p* value
Proteinuria						
Insignificant	10 (17.2)	45 (77.6)	3 (5.2)	58 (100.0)	Fisher's	1.000
Significant	0 (0.0)	2 (100.0)	0 (0.0)	2 (100.0)		
Haematuria						
Insignificant	10 (17.9)	43 (76.8)	3 (5.3)	56 (100.0)	Fisher's	1.000
Significant	0 (0.0)	4 (100.0)	0 (0.0)	4 (100.0)		

**Table 7:** Correlation of eGFR with frequency of crises in HbSS subjects.

Variable	eGFR	
	*r*	*p*
Frequency of blood transfusion	0.679	≤0.001
Frequency of painful crisis	0.138	0.295
Haemoglobin concentration	-0.147	0.264

## Data Availability

The data used to support the findings of this study are included within the article.
